# The time has come to implement routine outcome monitoring in mental health services across Latin America

**DOI:** 10.3389/fpubh.2025.1557029

**Published:** 2025-05-14

**Authors:** Clara Paz, Denisse Dogmanas, Alex Behn

**Affiliations:** ^1^Grupo de Investigación en Bienestar, Salud y Sociedad, Escuela de Psicología y Educación, Universidad de Las Américas, Quito, Ecuador; ^2^Facultad de Psicología, Universidad de la República, Montevideo, Uruguay; ^3^Escuela de Psicología, Pontificia Universidad Católica de Chile, Santiago, Chile; ^4^Instituto Milenio para la Investigación en Depresión y Personalidad, Santiago, Chile

**Keywords:** routine outcome monitoring, mental health services, digital data collection, data-driven decision-making, outcome measures, practice-based evidence

## Abstract

This perspective highlights the transformative potential of routine outcome monitoring in mental health care and public health, advocating for its adoption in Latin America to enhance data-driven decision-making and service quality across the lifespan. The discussion examines global advancements alongside local efforts to implement routine outcome monitoring, addressing key challenges such as infrastructure limitations, clinician engagement, and the need for contextual adaptations. Central to these efforts are strategies like utilizing digital platforms, fostering political commitment, securing financial investments, and prioritizing person-/patient-centered approaches. By integrating routine outcome monitoring into public health systems, policymakers and practitioners can better monitor mental health trends, allocate resources effectively, and design interventions that address community needs. While significant barriers remain, we urge the adoption of digital routine outcome monitoring solutions in Latin America and call for collaborative efforts to fully realize the potential of routine outcome monitoring in improving both mental health services and broader public health initiatives.

## 1 Introduction

Routine Outcome Monitoring (ROM) refers to the practice of systematically tracking patient progress throughout treatment using standardized measures that map relevant symptoms or areas of dysfunction targeted by interventions (1). ROM is a crucial component of measurement-based care in mental health. ROM allows for implementing data-driven and personalized clinical decision-making processes (2), ongoing feedback to therapists about key variables, and systematic service quality assessment (3).

A relatively large and growing body of research has been devoted to ROM, specifying its merits and different areas of application (4). However, most of the literature on mental health evaluation processes comes from high-income countries; in middle- and low-income countries, the ROM of mental health data is generally poor or non-existent (5). The lack of rigorous evaluation of mental health programs poses a significant barrier to their acceptance and expansion, contributing to the gap in health services (6). Despite the challenges in implementing ROM, observed even in high-income settings, the time has come to advocate for the progressive implementation of ROM in mental health in Latin America (LA). This can benefit from a growing body of research and more affordable and accessible implementation technologies (e.g., ubiquitous smartphone use).

Data collection of process and outcome measures has been highly recommended by the World Health Organization (7). Worldwide, multiple countries strive to implement systems for evaluating and measuring healthcare quality. Roe et al. (8) reviewed strategies in various countries from 2000 to 2018 concerning patient-reported outcome measurements and routine outcome measures in mental health. They found that 15 countries have programs with these measures in different stages of development, concluding that there is great variability in ROM models across countries. In the absence of strong nationwide policy efforts to support routine evaluation practices and strategies, implementation amounts to scattered and typically short-lived efforts that do not capitalize on the benefits associated with ROM. There are, however, prominent international examples where nationwide directives have facilitated the implementation of ROM.

Since 2000, Australia has mandated the use of outcome measures for all mental health service users. Public mental health clinicians must collect data upon admission, discharge, and at regular intervals (9). Outcome measurement serves various purposes in the country: monitoring quality and effectiveness, guiding clinical decisions, aiding discharge planning, improving treatment engagement, tracking user progress, evaluating service delivery models, and informing system-wide reforms for policymakers and planners.

In the UK, the government has mandated the routine use of outcome measures (10). Mental health organizations contribute data for comparison via the National Health Service Benchmarking Network. Outcome measurement is increasingly integrated into commissioning and service quality assurance processes. Additionally, the Adult Improving Access to Psychological Therapies program, initiated in 2008, aims to enhance access to evidence-based treatments for depression and anxiety within the National Health Service (11). Data usage ensures equitable quality services, aiding clinicians and patients in tracking progress and fostering collaborative decision-making. Supervision and service monitoring utilize data for performance analysis, enhancing service quality, value, and outcomes.

One of the most recent endeavors is that promoted in the Netherlands (12). ROM has become a major focus, with the establishment of a centralized monitoring system overseeing reimbursed mental health interventions to improve care standards. The Foundation for Benchmarking Mental Health (SBG) serves as an autonomous knowledge center for mental health providers and insurance firms.

In Latin American (LA) there is a recognition of the importance of ROM in academia (13). Still, no country in the region has a currently implemented strategy at the national level. We conducted a systematic review of all National Mental Health plans and strategies in LA countries to determine whether the use of Routine Outcome Monitoring (ROM) is currently recommended by governments and/or ministries of health. Among the 20 LA countries (excluding insular LA), only seven—Argentina, Bolivia, Chile, Colombia, Ecuador, Paraguay, and Uruguay—had specific country-wide or department-level mental health plans, strategies, or guidelines. Of these, only the plans and guidelines from the governments of Chile and Uruguay included monitoring strategies aligned with ROM. These strategies specifically recommended measuring outcomes at the beginning and end of mental health treatments but did not advocate for session-by-session monitoring. Notably, Chile’s plans included recommendations for specific outcome measures to achieve this purpose.

## 2 Why should ROM be used?

Implementing systems to monitor outcomes and feed them back to clinicians and service users can improve engagement in treatment and may improve outcomes. Establishing a data stream via ROM proves advantageous at various levels (14).

At the direct care level, research shows that when ROM is adopted, accepted, and its benefits are explained to clinicians and patients, it can positively impact treatment outcomes (15). At the service level, ROM enhances care, streamlines procedures, and aids quality control. Aggregated ROM data across services can inform system-wide improvements, aiding policymaking based on patient, service, and system-level data (16).

Given the lifelong impact of mental health conditions, integrating ROM across all stages of care is essential, from early intervention in youth to support for older adults. It is particularly critical during adolescence and young adulthood, where demonstrating meaningful and relevant outcomes fosters trust and sustained engagement in care. Developing age-appropriate, culturally sensitive, and youth co-designed outcome measures is key to ensuring ROM effectively supports decision-making and enhances service quality (17).

ROM facilitates value-based clinical care in both private and public sectors, ensuring efficient resource allocation and delivering standardized interventions effectively and accountably. Given global prevalence estimates, it is particularly relevant in mental health care (18). ROM data can impact decision-making from the therapy room to statewide policy, improving outcomes and aiding clinical decision-making, resource allocation, training needs, and, ultimately, health policy.

Amidst the challenges posed by the COVID-19 pandemic, several health systems have struggled with service access and resource availability. Tracking patients progress offers valuable insights for treatment improvement; it provides evidence to stakeholders (e.g., patients, insurance companies, health system authorities), facilitates service comparisons and contributes data for research.

It is important to note that ROM goes beyond measurement, encompassing tracking mental health changes over time, interpreting trends, and adjusting treatment strategies accordingly. It is an iterative process that informs clinical decisions, ensuring the delivery of effective interventions tailored to individual needs.

## 3 What are the challenges, needs, and contextual adaptations of using ROM in LA?

Implementing ROM in mental health services faces common challenges worldwide (19). This process requires logistical, technical, and behavioral changes over several years, placing demands on organizational resources. Success hinges on active support from managers and authorities, who play a critical role in securing sustainable resources for ROM (20). However, a proactive, data-driven approach through ROM enhances patient outcomes, optimizes resource use, and results in more efficient, cost-effective mental health services (21). These benefits underscore the need to adopt ROM across LA.

While some nations have seen success through national-level ROM initiatives, engagement from local organizations remains a common challenge, often due to strategies that overlook regional diversity (22). The diversity within each country requires careful planning of aspects such as staff involvement, inter-organizational communication, and local engagement. While large cities may offer adequate and tailored services, smaller towns or communities often face significant gaps in access.

Within LA a varied range of social, economic, and political contexts influences how mental health services are delivered, especially in indigenous and remote communities. These groups may face unique access barriers, including reliance on traditional healing practices, linguistic differences, and a mistrust of state-run programs stemming from historical marginalization. Moreover, the geographic isolation of some areas limits the effectiveness of standardized models like ROM, demonstrating the need for tailored, context-sensitive approaches.

Implementation of ROM across LA is also complicated by differences in governance and local health infrastructure, which vary widely between and within countries. In some areas, robust local health systems with active community engagement facilitate smoother implementation. However, in under-resourced areas, these conditions hinder service delivery and complicate the adoption of new initiatives. Effective ROM implementation must adapt to these diverse layers—from national to local—to bridge disparities and meet community needs. Strategies that involve stakeholders at every level, incorporate community input, and respect cultural differences will foster equitable and effective outcomes.

Technological development presents a considerable challenge for the implementation of ROM in LA. Many countries in the region remain in the early stages of adopting data collection technologies, with significant disparities in infrastructure and access across urban and rural areas. Larger urban mental health services often have an advantage, as they are more likely to have the resources to integrate advanced ROM software, including stable internet, modern hardware, and trained technical personnel.

However, rural and underserved areas face persistent obstacles, such as unreliable internet connectivity, limited availability of digital devices, and a lack of technical support. Addressing these disparities calls for innovative, context-sensitive solutions. For instance, low-tech alternatives, like paper-based data collection tools or offline-capable platforms, can bridge the technological divide. Additionally, the growing accessibility of mobile health (mHealth) applications offers promising opportunities. These apps, equipped to function with intermittent internet access, allow data collection and storage that can later sync with centralized systems when connectivity is restored.

Community-driven and participatory strategies could further enhance ROM adoption in diverse settings. Collaborating with local stakeholders to identify technological needs and preferences can result in solutions that are both practical and sustainable. For example, training community health workers to use simplified mHealth tools or introducing SMS-based monitoring systems can ensure data is gathered effectively, even in remote areas (23).

Ultimately, addressing technological disparities requires a multi-tiered approach—combining high-tech ROM tools for urban centers with scalable, low-tech solutions for rural regions (24). Investments in infrastructure, expanded access to mobile technologies, and ongoing technical training will be essential to ensure equitable implementation of ROM across LA, unlocking its potential to transform mental health care delivery in both urban and underserved populations.

Patient characteristics, such as literacy levels, cognitive abilities, language proficiency, and culturally specific views of mental health, also present challenges to ROM adoption (25). Literacy and language diversity across Latin America make questionnaire completion challenging, and ROM measures often lack cultural adaptation for local populations. Tailoring ROM measures to reflect how distress is expressed within various cultural contexts—especially in indigenous communities where psychological issues are often described in physical or spiritual terms—enhances their relevance and effectiveness. Collaboration with bilingual and bicultural professionals and community input can ensure ROM tools resonate with local cultural meanings.

Finally, staff workload, limited training, and concerns about performance evaluation add to implementation challenges (20, 26). In Latin America, where training on ROM is rare, professionals may not fully understand its practical benefits (27, 28). The use of ROM to evaluate program quality and effectiveness offers a compelling reason for its adoption. However, to gain clinicians’ support, ROM should be framed as a tool for enhancing patient care rather than as a performance metric. Early involvement, user-friendly systems, and training can help secure clinician buy-in, minimizing the perception of ROM as merely an administrative task. Integrating clinicians’ perspectives is essential to tailoring ROM for practical use, improving its acceptance, and ensuring meaningful implementation. Despite all these challenges, some services in the region have already implemented ROM. Table 1 showcases three examples.

**Table 1:** Examples of ROM strategies implemented so far in Latin America.

Examples of ROM in Latin America		
A University Clinic in Quito, Ecuador	A Private Practice Service in Buenos Aires, Argentina	A University Medical Center in Santiago Chile
The Applied Psychology Center operates within the Universidad de Las Américas, Ecuador, offering mental health services to both students and the general population. Within this service, ROM is utilized for training, research, and enhancing service quality. Data is collected per session, and trainees are responsible for ensuring the completion of questionnaires and tracking the information using a web-based application (30). Patient information is leveraged for therapist supervision and service enhancement through discussions based on aggregated data (31). An experienced therapist, overseeing both therapists and trainees, highlights the utility of ROM: I believe that monitoring itself is crucial for mapping each process and observing its evolution from a broader perspective. Consequently, this offers us insight into each case and can even facilitate an understanding of individual clients’ specific processes, as well as provide a summary of all treated clients.	The Aiglé Foundation has committed itself to training and research for over 40 years, creating a psychotherapy program available to the public and initiating a community effort to aid low-income groups. Consistently, the institution receives consultation requests from people in Buenos Aires and nationwide. These individuals undergo an intake process and psychological assessment before being linked to a specialized network of professionals (32). After this intake process, some individuals start a routine monitoring of their outcomes. ROM is implemented in the first five sessions, then every two sessions until session 21, and subsequently every 4 sessions. ROM is utilized for feedback-informed treatment, with the data serving for intervention quality control and supervision of consenting patients undergoing monitoring. Apart from being used as a clinical tool to provide feedback to patients, ROM simultaneously serves to conduct practice-based research. In this sense, ROM is the basis of Aigle’s current research program and serves as an important transdiagnostic element that allows adjustments to aspects of the therapeutic process. A 30-year-old female therapist, part of the Aiglé Therapists Network, utilizing the Aiglé Integrative Model, suggests the following regarding ROM: “As a therapist, patient monitoring has been crucial, allowing me to develop reflective practice. Additionally, by providing information about therapeutic progress, it served as a tool for comparing and contrasting with my clinical judgment. The data obtained from monitoring allowed the possibility of reviewing treatment advances or stagnation with patients and making necessary adjustments. Personally, I believe that monitoring patients has benefited my professional practice.”	The Centro Médico San Joaquín (CMSJ) is a university medical center, with a large catchment area in the southern part of Santiago, the biggest city and capital of Chile. Since 2022 the Mental Health Information Reporting Assitant (MHIRA) (33) is being used in a ROM study at one of the specialty clinics at CMSJ, namely the Adult Psychotherapy Unit. For many years, this unit has tried to implement procedures to measure the psychotherapy progress of patients on a session-to-session basis. Before MHIRA was implemented, many attempts had failed because the procedure was conducted using pencil and paper forms. Since MHIRA was implemented, patients have been approached by clinic staff (undergraduate students) with a tablet at their first psychotherapy. To date, 167 patients have completed an overall number of 688 assessments on a session-to-session basis. ROM data is currently being used for research and training purposes. A young psychiatry resident at the CMSJ indicated that “Precision therapy (what is best for which patient at a given moment) is something that therapists develop empirically in their regular practice. ROM allows for more precise development of this, providing data, monitoring, and recommendations that have been shown to improve clinical practice and the therapeutic bond. As a physician by training, I see it as precision instruments such as a thermometer or a pulse oximeter that allow us to make better clinical decisions and deliver modern, tailored care to each user.”

## 4 General recommendations for the implementation of ROM in LA

The introduction of ROM in LA holds promise for enhancing the availability of local data and fostering data-driven decision-making. Considering the above-mentioned challenges, we offer recommendations to create a supportive environment for ROM implementation across the region. The cost of successful implementation of ROM strategies can vary significantly, depending on the scope and sophistication of the procedure being considered. For instance, a nationwide ROM protocol covering all mental health sessions offered to patients will cost significantly more than a ROM procedure implemented in a single specific health service. Strategies that can guide resource allocation, prioritization, and implementation of ROM in LA include:

1. Starting small within a stepped-care model: ROM procedures must be implemented incrementally, starting with a few services and progressing to regional and nationwide approaches. This strategy is also useful because early technical or ideological problems with ROM can be spotted and corrected. Piloting different systems (e.g., software for instrument delivery and data storage), different measures (e.g., broadband or trans-diagnostic screeners versus diagnosis-specific self-reports), and different frequency set-ups (baseline, pre-post, or session-to-session measurements) can be explored in this stepwise approach to ROM implementation. It is also important that early pilot implementations demonstrate consistently higher efficacy, transparency, and even cost-saving to justify further, more ambitious implementation strategies can be justified.
2. Integration and collaboration among providers: ROM should be implemented within a team-based approach where primary care providers, mental health specialists, and care managers work together to deliver comprehensive care. This model enhances the capacity of stepped-care settings across medical specialties to manage mental health conditions effectively, facilitating communication, coordination, and continuity of care.
3. Task-shifting and task-sharing: ROM implementation may result in new clinical and administrative behaviors that burden providers. Current technology can significantly reduce the human resources needed to implement and monitor ROM routines. Still, task-shifting involves delegating tasks to less specialized health workers, while task-sharing ensures that tasks are shared among team members to maximize efficiency. This approach is particularly useful in resource-limited settings to absorb clinical and administrative tasks related to ROM. Within the strategy, investing in training mental health professionals and non-professionals to use ROM tools effectively is essential. This includes training on data collection, interpretation, and integration into clinical practice.
4. Integration with existing healthcare systems: This requires collaboration with local health authorities and stakeholders to align ROM practices with national health policies and infrastructure.
5. Academic-public partnerships: Academic institutions offer the research capabilities to develop and validate culturally appropriate ROM tools, ensuring their effectiveness across diverse populations. Additionally, public health agencies provide the infrastructure and policy support necessary to integrate these tools into existing healthcare systems effectively. For instance, collaborative efforts in LA countries have demonstrated how academic-public health partnerships can enhance the implementation of ROM, thereby improving mental health outcomes and system efficiency.
6. Make a case for value-based mental healthcare: ROM is a cornerstone of value-based healthcare, which emphasizes delivering high-quality care that improves patient outcomes relative to costs. 28 ROM facilitates the systematic tracking of patient progress and treatment efficacy, allowing for real-time adjustments to care plans. This ensures that interventions are both effective and efficient. By aligning healthcare delivery with patient-centered outcomes and optimizing resource use, ROM supports the broader goals of value-based healthcare. This approach has enhanced overall health system performance and patient satisfaction in various healthcare settings.

Resource allocation must be accompanied by the promotion of professional commitment to ROM, emphasizing quality control through training to ensure accurate deployment. Clinicians’ buy-in is crucial, given their workload. Outcome monitoring should resonate with clinicians to prevent it from being seen as an administrative burden. Strategies to promote buy-in include early involvement, user-friendly systems, and ongoing training. Caution is needed to avoid viewing ROM as performance evaluations, which could lead to resistance. Incorporating end-users’ perspectives tailors implementations to patients’ needs, ensuring ROM systems are effective and clinician-friendly, supporting their integration into practice. ROM implementation must be accompanied by robust monitoring and evaluation mechanisms to track the progress and evaluate its impact on patient outcomes and service delivery. Evaluation strategies may include monitoring clinicians’ adherence to ROM tools, cost-effectiveness analysis, stakeholder satisfaction assessments, system integration evaluation, and tracking long-term outcomes. Partnering with academic institutions and research organizations is vital to generate local evidence and enhance ROM evaluation and research capacity, ensuring its sustainability and effectiveness in improving mental health care across the region.

## 5 Conclusion

The overarching goal of this article is to advocate for adopting ROM systems to enhance data-driven decision-making within national mental health systems across the region and throughout all stages of life. While our focus is on larger systems, similar principles may apply to institutions and specific service settings. Institutions may explore implementing direct individual feedback systems for therapists and patients, while considering associated costs, training needs, and overarching objectives carefully where resources permit.

ROM necessitates outcome measures, typically requiring adaptation for local use in psychometric studies. This is crucial if ROM information will be used for research or high-stakes decision-making. However, early adoption of ROM strategies should not be discouraged. Officially translated instruments or measures not yet fully adapted and studied for their psychometric properties can be used initially in combination with traditional clinical evaluation (29).

While the ideal scenario involves meeting all the recommended conditions, it is essential to recognize that practical implementation may not always align perfectly. Nevertheless, the time has come for ROM implementation in LA. Efforts should be directed toward this purpose, and creative solutions can be developed while the ideal scenario is established within the region’s different countries. Let us collectively work toward realizing the full potential of ROM and improving mental health care across LA for individuals at every stage of their lives.

## Data Availability

The original contributions presented in the study are included in the article/supplementary material, further inquiries can be directed to the corresponding author.
